# Food-related Quality of Life in patients with Inflammatory Bowel Disease: Translation and Validation of the German version of FR-QoL-29

**DOI:** 10.1055/a-2542-6781

**Published:** 2025-03-31

**Authors:** Lea Pueschel, Katharina Hupa-Breier, Heiner Wedemeyer, Henrike Lenzen, Miriam Wiestler

**Affiliations:** 1 9177 Department of Gastroenterology, Hepatology, Infectious Diseases and Endocrinology, Hannover Medical School, Hannover, Germany; 2 39726 Gastroenterologie und Diabetologie, Städtisches Klinikum Braunschweig gGmbH, Braunschweig, Germany

**Keywords:** IBD, Nutrition and metabolism, patient-reported outcome measures (PROMS), Inflammatory bowel disease, Quality of life, CED, Ernährung und Stoffwechsel, Patient Reported Outcomes (PRO), Chronisch entzündliche Darmerkrankungen, Lebensqualität

## Abstract

**Background and aims:**

The psychosocial effects of eating and drinking – summarized as food-related quality of life (FR-QoL) – are underexplored in inflammatory bowel disease (IBD). Currently, there is no German instrument to assess FR-QoL in IBD patients. This study aimed to translate the validated English FR-QoL-29 questionnaire into German and evaluate its validity and reliability.

**Methods:**

A monocentric, cross-sectional study was conducted at a tertiary referral center with IBD patients and healthy controls. Participants completed questionnaires on sociodemographics, disease history, the Malnutrition Universal Screening Tool (MUST), the German Short Health Scale (SHS), and the FR-QoL-29-German. The FR-QoL-29 was translated into German using a forward-backward method. Its reliability and validity was assessed using Pearson correlation coefficients, intraclass correlation coefficients, and Cronbach’s α.

**Results:**

N=200 IBD patients (Crohn’s disease: 61.8%; women: 50.8%; remission: 56.2%) and n=10 healthy controls completed the questionnaires. Overall, 113 IBD patients repeated the questionnaires after an average of six weeks. Significant differences in FR-QoL-29-German sum scores were found between all levels of IBD disease activity, except for remission – mild disease (p = 0.423) and moderate – severe disease (p = 0.999). FR-QoL-29-German scores significantly correlated with age (p = 0.041), disease activity (p < 0.001), MUST (p = 0.015), fecal Calprotectin (p = 0.011) and SHS (p < 0.001). Overall, the FR-QoL-29-German showed excellent internal consistency (Cronbach’s α = 0.965) and good test-retest reliability (ICC = 0.85 [95% CI: 0.78–0.89]).

**Conclusion:**

The FR-QoL-29-German is a valid and reliable tool for assessing food-related quality of life in German-speaking individuals with IBD.

## Zusammenfassung

**Einleitung:**

Die psychosozialen Auswirkungen des Essens und Trinkens – zusammengefasst als ernährungsbezogene Lebensqualität (FR-QoL) – sind bei chronisch entzündlichen Darmerkrankungen (CED) noch wenig erforscht. Derzeit gibt es kein deutsches Instrument zur Bewertung der FR-QoL bei CED-Patienten. Ziel dieser Studie war es, den validierten englischen Fragebogen FR-QoL-29 ins Deutsche zu übersetzen und seine Validität und Reliabilität zu bewerten.

**Methodik:**

Eine monozentrische Querschnittsstudie wurde an einem tertiären Referenzzentrum mit CED-Patienten und gesunden Kontrollpersonen durchgeführt. Die Teilnehmer füllten Fragebögen zu soziodemografischen Daten, zur Krankheitsgeschichte, zum Malnutrition Universal Screening Tool (MUST), zur deutschen Short Health Scale (SHS) und zum FR-QoL-29-German aus. Der FR-QoL-29 wurde mithilfe einer Vorwärts-Rückwärts-Methode ins Deutsche übersetzt. Seine Reliabilität und Validität wurde anhand von Pearson-Korrelationskoeffizienten, Intraklassen-Korrelationskoeffizienten und Cronbachs α bewertet.

**Ergebnisse:**

N=200 CED-Patienten (Morbus Crohn: 61,8%; Frauen: 50,8%; Remission: 56,2%) und n=10 gesunde Kontrollpersonen füllten die Fragebögen aus. Insgesamt 113 CED-Patienten wiederholten die Fragebögen nach durchschnittlich sechs Wochen. Signifikante Unterschiede in den FR-QoL-29-German-Summenwerten wurden zwischen allen Stufen der CED-Krankheitsaktivitäten gefunden, mit Ausnahme von Remission – milde Erkrankung (p = 0,423) und moderate – schwere Erkrankung (p = 0,999). Die FR-QoL-29-German-Summenwerte korrelierten signifikant mit dem Alter (p = 0,041), der Krankheitsaktivität (p < 0,001), MUST (p = 0,015), fäkalem Calprotectin (p = 0,011) und SHS (p < 0,001). Insgesamt zeigte der FR-QoL-29-German eine ausgezeichnete interne Konsistenz (Cronbachs α = 0,965) und eine gute Test-Retest-Reliabilität (ICC = 0,85 [95% CI: 0,78–0,89]).

**Schlussfolgerung:**

Der FR-QoL-29-German ist ein valides und zuverlässiges Instrument zur Erfassung der ernährungsbezogenen Lebensqualität bei deutschsprachigen CED-Patienten.

## Introduction

Inflammatory bowel disease (IBD), in particular the two main clinical phenotypes, ulcerative colitis (UC) and Crohn’s disease (CD), are characterized by recurrent, chronic inflammation of the gastrointestinal tract. IBD behavior seems to be closely linked to nutrition with the latest research indicating that diet and nutrition are significantly involved in the etiopathogenesis of the disease, although their specific role throughout its clinical course remains unclear 1 2 . In addition, patient reports show a subjective connection between disease behavior and nutrition 3 4 , while IBD related quality of life seems to be connected to patients’ dietary behavior 5 . Along with a perceived treatment frustration some patients hence turn to online advice and to extreme nutritional measures 6 . In fact, in patients with IBD, there are many drivers for dietary change that are also considered risk factors for disordered eating – with IBD patients exhibiting many of the confounding traits commonly seen in patients with eating disorders, including anxiety, depression, self-initiated diets, poor body image and/or shame (particularly those with ostomy) 7 8 . It is known from the general population that quality of life (QoL) can be associated with food and nutrition 9 10 . In addition, previous studies show that this is in particular true in possible food-triggered disease such as celiac disease or food-based allergies 11 12 . Consequently, the latest research is now focusing on IBD food-related QoL 13 . However, to identify possible needs for physician-intervention validated patient reported outcome measures (PROMs) assessing food related quality of life are needed. The FR-QoL-29 was developed based on qualitative interviews 14 and is a validated PROM that queries self-imposed dietary restrictions as well as impairments in daily life due to eating and drinking 15 and has successfully been used in English-speaking countries. Subsequently, it has already been translated and validated into Turkish 16 and Portuguese 17 . Meanwhile, there is no comparable validated tool in the German-speaking area. However, German-speaking patients and healthcare providers would truly benefit from this tool in both research and clinical practice.

## Materials and methods

### Ethical considerations

This monocentric study was approved by the ethics committee of Hannover Medical School (10847_BO_S_2023), registered at the German Clinical Trial Register (DRKS) as DRKS00032771 and the study design is in accordance with the Declaration of Helsinki (2013).

### Participants and setting

Between October 2023 and August 2024, a total of 200 IBD patients as well as 10 healthy controls were enrolled at the IBD outpatient clinic of Hannover Medical School. Prior to study inclusion, each patient was required to provide written informed consent. Eligibility criteria for study inclusion were a confirmed diagnosis of either UC or CD and disease duration of at least three months. Individuals with any disorders that preclude the assessment of the nature, scope, and potential consequences of the study were excluded.

### Data sources/measurements

All individuals who gave informed written consent were asked to complete an online demographic survey including data on sex and gender identity, body status (weight, height), age, marital status, employment status, and more. The survey included further questions pertaining to IBD-specific history, therapies, surgical history, and comorbidities. The degree of disease activity was determined in investigator-led interviews using either the German version of the Harvey-Bradshaw Index (HBI) 18 for CD patients or the German version of the partial Mayo score (PMS) 19 for those with UC. The extent of the disease was determined using the Montreal Classification for patients with CD and the anatomic extent for patients with UC 20 .

### FR-QoL-29-German

The FR-QoL-29 was translated into German with explicit permission granted by the copyright holders (Prof. Kevin Whelan, King’s College London). The questionnaire was translated via the forward-backward translation method 21 taking into account the special context of a cross-cultural adaptation 22 23 , which saw two translators who are fluent in English translate the questionnaire independent from each other. Both translations were then reviewed, and any discrepancies were resolved jointly by the research team, consisting of gastroenterologists, IBD experts, nutritionists, a linguist and the translators. The questionnaire was then translated back into English by a native English speaker who was fluent in German, not familiar with the English version, and not affiliated with the research team. A Pre-test was conducted with five IBD patients who, after having completed the translated questionnaires, were asked to evaluate the FR-Qol-29-German version in investigator-led interviews. Based on this feedback the research team finalized the FR-Qol-29-German version. The original questionnaire, as well as the German version, consists of 29 statements on food-related quality of life in the last two weeks. For each statement, one of five possible scored answers can be chosen, ranging from *Strongly agree (1)* to *Strongly disagree (5)* . Statements 8, 9, 24 and 25 are expressed in the positive, therefore the corresponding answers must be inverted when evaluating the questionnaire. Overall, the FR-QoL-29 score ranges from 29 to 145, with 145 indicating a good IBD food-related quality of life 15 .

### Malnutrition Universal Screening Tool

The German version of the Malnutrition Universal Screening Tool (MUST) was used to identify adult individuals with potential risks of malnutrition 24 25 . The MUST scoring is based on three areas: a) body mass index, b) unplanned weight loss within the last 3 to 6 months, c) acute illness with an expected food abstinence of at least five days. A maximum of 2 points can be awarded per area and a total of 0 to 6 points can be scored, with 0 points indicating a low risk and anything above two points suggesting a high individual risk of malnutrition.

### Short Health Scale

The validated German version of the Short Health Scale (SHS) 26 was used to assess the current IBD-related health status via four questions about symptoms, daily activities, disease-related concerns, and general well-being over the past seven days. Evaluation of all four domains allows for a maximum score of 400 points with higher scores indicating poorer health-related quality of life for individuals with IBD.

### Disease activity

Disease activity and remission were determined using entity-specific disease activity index cutoffs. For binary disease activity assessment remission was defined as a Harvey-Bradshaw Index (HBI) of <5 18 or a partial mayo score (PMS) of 0–1 19 , respectively.

### Statistical analyses

For statistical analysis SPSS Statistics software, version 28.0.1.0 (SPSS, IBM, Armonk, NY), and GraphPad PRISM, version 10.3.0 (GraphPad Software, Boston, Massachusetts, USA) were employed. Normal distribution was assessed via Shapiro-Wilk test. Categorical baseline variables are expressed as total and percentage. Significance levels are two-sided if not mentioned otherwise, and when applicable clinical relevance is reported as effect size estimate (d).

### Validity

Content validity of the translated FR-QoL-29-German items was assessed based on the expert judgment method 27 as well as the investigator-led pre-test interviews. Meanwhile, to assess construct validity, we demonstrated convergent and discriminant validity via hypothesize testing and exploratory factor analysis (EFA). We hypothesized a negative correlation between FR-QoL-29-German and a) health-related QoL in IBD individuals measured by SHS, and b) malnutrition risk score measured by MUST. We further hypothesized that we would see significant differences in FR-QoL-29-German scores for individuals in remission compared to those with an active disease, in addition we proposed that the Fr-QoL-29-German discriminates between IBD patients and healthy controls. For hypothesis testing we used student’s t-test and ANOVA for comparisons of the FR-QoL-29-German score between groups, and Pearson correlation coefficient to assess correlation between IBD health-related quality of life with IBD food-related quality of life. Data adequacy for factor analysis was assessed via Kaiser-Meyer-Olkin (KMO) test and Bartlett’s test of sphericity.

### Reliability

Internal consistency for the FR-QoL-29-German was assessed via Cronbach’s α while the reliability of the translated questionnaire was investigated by inviting all study subjects to complete a follow-up questionnaire, with n=113 completing the follow-up questionnaire before data analysis cutoff. Pearson correlation coefficient and intraclass correlation coefficient (ICC) for FR-QoL-29-German scores were used to assess test-retest reliability. On average the follow-up questionnaire was completed 6 weeks after study inclusion. Testing conditions for the FU-Questionnaire did not differ from the initial run as both the baseline survey as well as the follow-up questionnaire were conducted online. Patients with major changes in symptom characteristics (n=6) were excluded from the follow up analysis. As an additional quality criterion, we tested for possible ceiling and floor effect of the FR-QoL-29-German 28 .

### Sampling strategy

To eliminate distortion when comparing FR-QoL-29-German scores of IBD patients with those of healthy controls a random sample of the IBD cohort was drawn that matched the healthy control cohort in a ratio of 4:1.

## Results

### Study population

A total of 200 IBD patients were included in this study. In total, n=113 patients completed the FU questionnaire excluding those who had major changes in symptom characteristics since baseline (n=6). Baseline characteristics showed a skewed distribution of disease entities (Crohn’s disease 61.8%), but a balanced distribution of sex (women: 50.8%) and dichotomous remission status (remission: 56.2%). Overall, 45% of patients reported having received nutritional counseling at least once in the past due to IBD. Patients had a mean age of 40 years and a mean BMI of 25.25 kg/m ^2^ ( Table 1 ). For hypothesis-testing we matched a random IBD sample (n=40) to healthy controls (n=10; women = 60%; age = 36±5).

**Table:** **Table 1** Baseline characteristics. Variables are expressed as total and percentage (n[%]) or mean and standard deviation (M±SD). MUST – Malnutrition Universal Screening Tool; BMI – Body Mass Index; UC – Ulcerative Colitis.

		Baseline (n=200)
Demographics [Mean±SD or n(%)]		
Disease entity	Crohn’s disease	123 (61.8%)
	Ulcerative colitis	76 (38.2%)
Female		101 (50.8%)
Disease Activity	Remission	104 (56.2%)
	Mild Disease	46 (24.9%)
	Moderate Disease	28 (15.1%)
	Severe Disease	7 (3.8%)
Location of Crohn’s	L1	35 (28.2%)
	L2	21 (16.9%)
	L3	67 (54%)
	L4	13 (10.5%)
Crohn’s behavior	B1	46 (37.1%)
	B2	54 (43.5%)
	B3	24 (19.4%)
	perianal disease	33 (26.6%)
UC Montreal classification	Proctitis	5 (6.6%)
	Left-sided colitis	26 (34.2%)
	Pancolitis	45 (59.2%)
MUST	Low Risk	114 (57%)
	Medium Risk	40 (20%)
	High Risk	46 (23%)
Disease duration in years		14±10
Nutritional counselling in the past due to IBD		90 (45%)
FR-QoL-29-German score		84±22
Short Health Scale score		179±114
Gastrointestinal surgery		66 (34%)
BMI (kg/m ^2^ )		25.25±5.48
Age (years)		40±14
Smoking status (current or former)		73 (36.7%)
Calprotectin (mg/kg)		740.47±1727.6
C-reactive protein (mg/l)		5.98±13.18

### Translation and cross-cultural adaptation

The translation and subsequently cross-cultural adaptation process of the FR-QoL-29-German consisted of several steps and was at least partially carried out blind. Despite changes in sentence structure as well as the use of cultural appropriate synonyms of selected words, the final translation is in effect analogues to the original. However, similar to the FR-QoL-29-Portuguese 17 , the labeling of the Likert scale was also discussed in the German translation and no literal translation was chosen for *Strongly agree (1)* and *Strongly disagree (5)* , but rather the German expression *Stimme voll und ganz zu (1)* (English translation: *Completely agree (1)* ) and *Stimme überhaupt nicht zu* (English translation: *Do not agree at all (5)* ), which are frequently used in German standardized questionnaires.

### Validity

**Fig. 1:**
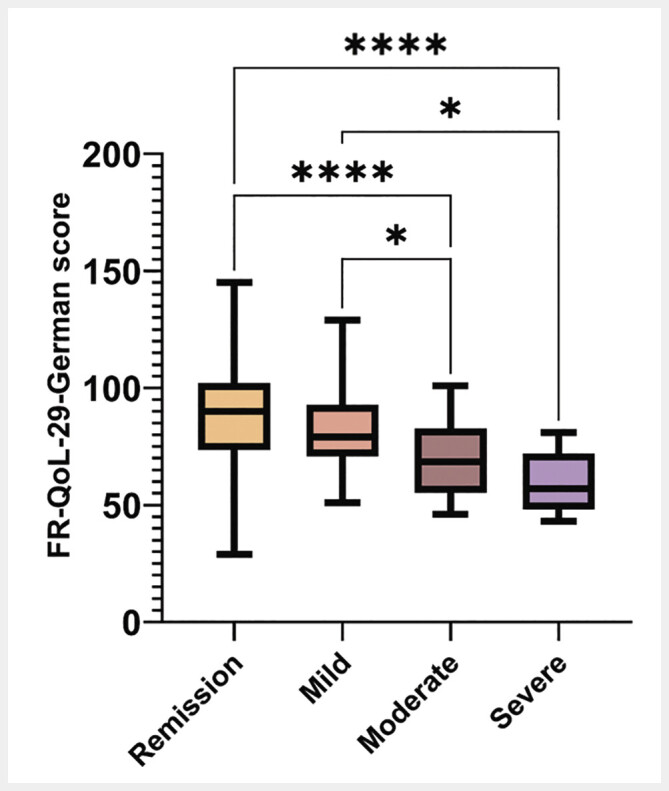
Comparison of FR-QoL-29-German score between entity-specific disease activity. Bonferroni correction of one-way ANOVA showed statistically significances of mean FR-QoL-29-German score between remission and severe disease (p < 0.001); remission and moderate disease (p < 0.001); mild and moderate disease (p = 0.035); mild and severe disease (p = 0.021).

**Fig. 2:**
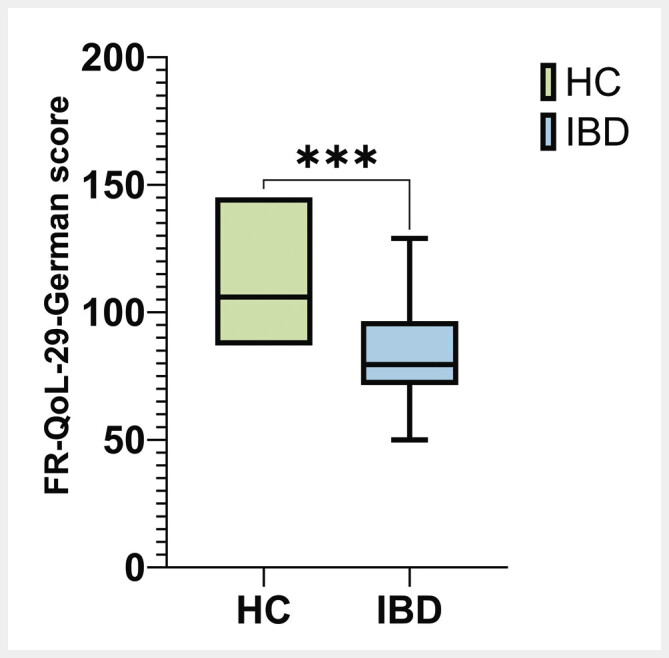
Matched comparison of FR-QoL-29-German score between healthy controls and IBD patients. Student’s t-test showed statistically significances of mean FR-QoL-29-German score between healthy controls and a matched subsample of IBD patients (p = 0.005; d = −1.4). HC – healthy controls; IBD – inflammatory bowel disease.

Requirement for factor analysis was satisfied with the inclusion of at least 145 enrolled patients which was further confirmed by the results of the Kaiser-Maier-Olkin test (0.95). Furthermore, Bartlett’s test of sphericity was significant (χ2 = 4423; p <0.001) thus indicating that the data is suitable for exploratory factor analysis (EFA). EFA identified four factors with an eigenvalue > 1 that accounted for a total variance of 65.63 %. However, a total of 51.41% of the variance was attributable to factor 1, therefore only factor 1 was retained ( Table 2 ). This is in accordance with the FR-QoL-29 15 as well as the FR-QoL-29-Turkish 16 . Hypothesis-testing for the hypotheses formulated in advance showed a negative correlation between FR-QoL-29-german score and a) SHS score (r = −0.502; p <0.001), and b) MUST score (r = −0.172; p = 0.008). Patients in remission scored significantly higher on the FR-QoL-29-German than patients with an active disease (p<0.001, d = 0.6). Bonferroni post-hoc test of one-way ANOVA revealed statistical significances of FR-QoL-29-German scoring between all entity-specific gradations of disease activity except for remission – mild disease (p = 0.423), and moderate disease – severe disease (p = 0.999) ( Fig. 1 ). One-tailed student’s t-test of FR-QoL-29-German score between healthy controls (n=10) and a matched random IBD sample (n=40) was statistically significant (p = 0.005; d = −1.4) ( Fig. 2 ). There was no statistically significant difference in FR-QoL-29-German score between disease entities (p = 0.198; d = −0.2). Correlation of demographic, health and disease-specific factors saw a positive correlation between FR-QoL-29-German score and age (r = 0.145; p = 0.041) as well as inverse correlations for disease activity (r = −0.303; p <0.001), MUST score (r = −0.172; p = 0.015), SHS score (r = −0.502; p <0.001), and fecal calprotectin mg/kg (r = −0.204; p = 0.011) ( Table 3 ). No ceiling or floor effect was apparent as out of 200 individuals with IBD only one (0.5%) scored 29 points thus indicating a very poor food-related quality of life, while one individual (0.5%) achieved the highest score of 145.

**Table:** **Table 2** Exploratory factor analysis for items of the German version of the FR-QoL-29.

FR-QoL-29-German item	Question	Factor 1
1	I have regretted eating and drinking things which have made my IBD symptoms worse	0.675
2	My enjoyment of a particular food or drink has been affected by the knowledge that it might trigger my IBD symptoms	0.621
3	My IBD has meant that I have had to leave the table while I am eating to go to the toilet	0.664
4	I have not been able to predict how long it will take for my body to respond to something I have had to eat or drink due to my IBD	0.740
5	Certain foods have triggered symptoms of my IBD	0.631
6	My IBD has meant that I have been nervous that if I eat something I will need to go to the toilet straight away	0.750
7	I have avoided having food and drink I know does not agree with my IBD	0.613
8	I have felt relaxed about what I can eat and drink despite my IBD	0.692
9	I have felt in control of what I eat and drink in relation to my IBD	0.585
10	I have struggled to eat the way that is best for my IBD because of other commitments during the day	0.544
11	I have been frustrated about not knowing how food and drink will react with my IBD	0.777
12	I have had to concentrate on what I have been eating and drinking because of my IBD	0.833
13	I have been worried that if I eat I will get symptoms of my IBD	0.846
14	I have felt the way that I eat and drink for my IBD has affected my day to day life	0.855
15	The way I have had to eat for my IBD has restricted my lifestyle	0.827
16	I have had to concentrate on what food I buy because of my IBD	0.751
17	It has been on my mind how my IBD will be affected by what I eat and drink	0.706
18	My IBD has prevented me from getting full pleasure from the food and drink I have had	0.805
19	I have felt that I need to know what is in the food I am eating due to my IBD	0.587
20	I have felt that I have had to be careful about when I have eaten because of my IBD	0.818
21	I have had to be more aware of what I am eating due to my IBD	0.771
22	I have missed being able to eat or drink whatever I want because of my IBD	0.872
23	I have felt that I would like to be able to eat and drink like everyone else	0.757
24	I have been happy to eat and drink around people I do not know despite my IBD	0.455
25	I have felt that I have been eating and drinking normally despite my IBD	0.615
26	I have found it hard not knowing if a certain food will trigger IBD symptoms	0.684
27	My IBD has meant I have had to make an effort to get all the nutrients my body needs	0.637
28	I have felt that I have not known how my IBD will react to food or drink	0.711
29	My IBD has meant that I have had to work hard to fit my eating habits in around my activities during the day	0.760
Eigenvalue		14.90
% of Variance		51.41

**Table:** **Table 3** Correlation of demographic, health and disease-specific factors with the FR-QoL-29-German score. BMI – Body Mass Index; MUST – Malnutrition Universal Screening Tool; SHS – Short Health Scale.

Correlation factors	Pearson correlation (r)	p
Age	0.145	**0.041**
Sex	0.125	0.078
BMI	0.072	0.313
Entity	0.093	0.189
Disease activity	−0.303	**<0.001**
MUST	−0.172	**0.015**
SHS	−0.502	**<0.001**
Calprotectin mg/kg	−0.204	**0.011**

### Reliability

Internal consistency was high with a Cronbach’s α coefficient of 0.965, in addition Cronbach’s α coefficient ranged from 0.962 to 0.966 if individual items were deleted. Pearson correlation coefficient showed good test-retest reliability between the initial FR-QoL-29-German score and the follow up FR-QoL-29-German score (r = 0.734, p <0.001) as did the calculated intraclass correlation coefficient of 0.85 [95% CI: 0.78; 0.89]. Single item correlation between initial and follow up timepoint were significant for all FR-QoL-29-German items (p<0.001) ( Table 4 ).

**Table:** **Table 4** Results for internal consistency analysis and test-retest reliability.

FR-QoL-29-German item	Corrected-item-total score correlation	Cronbach’s Alpha if item deleted	Test-retest reliability
1	0.658	0.964	0.574*
2	0.600	0.964	0.440*
3	0.641	0.964	0.508*
4	0.723	0.963	0.440*
5	0.613	0.964	0.490*
6	0.728	0.963	0.656*
7	0.589	0.964	0.550*
8	0.643	0.964	0.530*
9	0.532	0.965	0.508*
10	0.523	0.965	0.508*
11	0.757	0.963	0.542*
12	0.818	0.963	0.580*
13	0.830	0.963	0.584*
14	0.838	0.963	0.714*
15	0.807	0.963	0.638*
16	0.730	0.963	0.577*
17	0.683	0.964	0.526*
18	0.787	0.963	0.647*
19	0.561	0.965	0.599*
20	0.799	0.963	0.640*
21	0.749	0.963	0.527*
22	0.856	0.962	0.694*
23	0.734	0.963	0.515*
24	0.406	0.966	0.443*
25	0.560	0.965	0.543*
26	0.659	0.964	0.329*
27	0.612	0.964	0.478*
28	0.686	0.964	0.424*
29	0.737	0.963	0.405*
	**No of Items**		29
	**Cronbachs Alpha**		0.965
	**Intraclass correlation coefficient [95% CI]**		0.85 [95%CI: 0.78; 0.89]

## Discussion

The present study introduces a novel German translation of the FR-QoL-29, which has been developed for the systematic assessment of food-related quality of life in patients with IBD. Psychological distress and restricted food intake are associated with poorer health-related quality of life in IBD and other gastrointestinal diseases. However, in IBD, less is known about the relationship between mental health, restricted food intake, and food-related quality of life (FR-QoL), which directly measures the psychosocial impact of eating and drinking 14 29 30 . Patients with IBD report that nutrition is the most important psychosocial need affected by IBD 29 30 31 . Furthermore, food and diet are primary behavioral factors that can be used to help patients control the disease and symptoms 32 33 34 . However, because the role of diet and specific food components in disease development and pathogenesis remains unclear and the evidence base for nutritional therapies is limited, nutritional counseling is often inadequate 2 35 36 . The FR-QoL-29 is an important patient reported outcome measure (PROM) and invaluable in assessing food-related quality of life for IBD patients in terms of psychosocial factors. IBD-specific nutritional strategies in order to avoid a flare-up may be restricting or excluding certain foods. However, disease-related restrictive eating can be isolating, burdensome on everyday life, and exacerbate a decrease in mental health. Indeed, variations in eating behavior is not uncommon in IBD patients as fear of symptoms from eating is one major issue and studies have further shown that IBD patients are at high risk for disordered eating 37 38 . Our study has revealed a concerning percentage of individuals with a medium (20%) and high risk (23%) of malnutrition, which could exacerbate disease development 39 . The need for measures such as the FR-QoL-29 is therefore evident, however so far there has been no validated German translation. Using the forward-backward translation method 21 we subsequently assessed validity and reliability of the German translation of the FR-QoL-29 in an ongoing single-center cohort-study. Internal consistency of the FR-QoL-29-German was excellent with a Cronbach’s α coefficient of 0.965 and thus similar to the FR-QoL-29 (Cronbach’s α coefficient 0.959) 15 , the FR-QoL-29-Turkish (Cronbach’s α coefficient 0.96) 16 , and FR-QoL-29-Portuguese (Cronbach’s α coefficient 0.966) 17 . No items were excluded since Cronbach’s α coefficient was never higher than 0.966 after individual item deletion. By comparing the IBD sum score with the HC sum score, good discriminant validity was demonstrated. In addition, it was shown that the total score differs significantly between entity-specific gradations of disease activity, this is in accordance with the original publication. Consequently, when considered collectively, the FR-QoL-29 has been demonstrated to be consistent with health-related quality of life and disease activity surrogates in IBD.

We acknowledge that this study has certain limitations, including its monocentric design within a tertiary referral center, which may have introduced a degree of selection bias. Another potential limitation is the outpatient setting. It would be beneficial for future research to include hospitalized IBD patients with severe disease activity, in order to gain further insight into the deterioration in food-related quality of life in this distinct setting. In the present study, only seven patients with severe disease activity were included. However, as the majority of IBD patients are treated within an outpatient setting, this study represents a “real world cohort”. The present study has additional notable strengths. Firstly, the sample size of IBD patients was large and diverse. Secondly, the inclusion of healthy controls allowed for a more comprehensive comparison of the instrument’s performance.

In conclusion, the results of this study demonstrate that the FR-QoL-29-German is a valid and reliable tool for the assessment of food-related quality of life in German-speaking IBD patients. As there is an increasing body of evidence indicating a close relation between IBD and nutrition, a specific measure of the psychosocial aspects of eating and drinking in IBD enables physicians and other health-workers to assess the disease impact on patients’ food-related quality of life in a standardized way. This provides further insight into the significant impact of IBD on the daily lives of individuals. Further research is required to elucidate the explicit role and connection between patients’ food-related quality of life and the course of their IBD disease e. g. in terms of therapy-response or disease complications. We hereby present a tool for the systematic measurement of this aspect in German-speaking IBD patients.
